# Simotang Alleviates the Gastrointestinal Side Effects of Chemotherapy by Altering Gut Microbiota

**DOI:** 10.4014/jmb.2110.10018

**Published:** 2022-02-28

**Authors:** Lijing Deng, Xingyi Zhou, Zhifang Lan, Kairui Tang, Xiaoxu Zhu, Xiaowei Mo, Zongyao Zhao, Zhiqiang Zhao, Mansi Wu

**Affiliations:** 1 Guangzhou Key Laboratory of Formula-Pattern of Traditional Chinese Medicine, School of Traditional Chinese Medicine, Jinan University, Guangzhou 510632, P.R. China; 2 Hubei University of Chinese Medicine, Wuhan 430065, P.R. China; 3 School of Traditional Chinese Medicine, Beijing University of Chinese Medicine, Beijing 100029, P.R. China; 4 Department of Musculoskeletal Oncology, The First Affiliated Hospital of Sun Yat-sen University, Guangzhou 510080, P.R. China

**Keywords:** Simotang oral liquid, cisplatin, gastrointestinal side effects, gut microbiota

## Abstract

Simotang oral liquid (SMT) is a traditional Chinese medicine (TCM) consisting of four natural plants and is used to alleviate gastrointestinal side effects after chemotherapy and functional dyspepsia (FD). However, the mechanism by which SMT helps cure these gastrointestinal diseases is still unknown. Here, we discovered that SMT could alleviate gastrointestinal side effects after chemotherapy by altering gut microbiota. C57BL/6J mice were treated with cisplatin (DDP) and SMT, and biological samples were collected. Pathological changes in the small intestine were observed, and the intestinal injury score was assessed. The expression levels of the inflammatory factors IL-1β and IL-6 and the adhesive factors Occludin and ZO-1 in mouse blood or small intestine tissue were also detected. Moreover, the gut microbiota was analyzed by high-throughput sequencing of 16S rRNA amplicons. SMT was found to effectively reduce gastrointestinal mucositis after DDP injection, which lowered inflammation and tightened the intestinal epithelial cells. Gut microbiota analysis showed that the abundance of the anti-inflammatory microbiota was downregulated and that the inflammatory microbiota was upregulated in DDP-treated mice. SMT upregulated anti-inflammatory and anticancer microbiota abundance, while the inflammatory microbiota was downregulated. An antibiotic cocktail (ABX) was also used to delete mice gut microbiota to test the importance of gut microbiota, and we found that SMT could not alleviate gastrointestinal mucositis after DDP injection, showing that gut microbiota might be an important mediator of SMT treatment. Our study provides evidence that SMT might moderate gastrointestinal mucositis after chemotherapy by altering gut microbiota.

## Introduction

Chemotherapy is one of the three most commonly used methods for treatment of malignant tumors. Though chemotherapy is effective in killing cancer cells and preventing cancer from invasion and metastasis, patients receiving chemotherapy might also face various side effects. The toxicity and side effects caused by chemotherapy drugs are mostly gastrointestinal reactions, of which gastrointestinal mucositis is the most common [1]. The clinical manifestations of gastrointestinal mucositis include nausea, anorexia, vomiting, ulcers, abdominal pain, abdominal distension, diarrhea, constipation, and infection and could seriously affect daily life and even hinder the process of normal treatment of cancer patients. Therefore, drugs that are used to treat gastrointestinal mucositis are also used during cancer treatment to alleviate chemotherapy gastrointestinal side effects. Nowadays, the most commonly used anti-gastrointestinal side-effect drugs in clinical a clinical setting are antiemetics, with some examples being 5-hydroxytryptamine (5-HT) receptor antagonists (*e.g.*, palonosetron), neurokinin (NK) 1 receptor antagonists (*e.g.*, aprepitant), corticosteroids (*e.g.*, dexamethasone) and dopamine (DA) D2 receptor antagonists (*e.g.*, metoclopramide). However, these drugs might also be accompanied by side effects, for example, palonosetron might induce headaches and constipation [2, 3]. Aprepitant might cause headache, fatigue, neutropenia, constipation, and pruritus [4]. Dexamethasone might bring about glucose tolerance alteration, behavioral and mood changes [5]. And metoclopramide could lead to tardive dyskinesia, drowsiness and acute dystonic reaction [6]. Meanwhile, nutrition therapy can relieve the gastrointestinal side effects of patients receiving chemotherapy. For example, acidic foods such as maybush and orange might improve nausea and anorexia. Eating more dietary fiber could promote intestinal peristalsis and alleviate abdominal distension. Nutrition therapy generally does relieve mild gastrointestinal side effects of chemotherapy, but it does not help with more serious gastrointestinal side effects. It is necessary to develop new drugs that have both high efficacy and low toxicity to treat gastrointestinal side effects caused by chemotherapy.

Traditional Chinese medicine (TCM) has a history of more than 2,000 years and has cured countless numbers of patients over the centuries. For example, Youyou Tu discovered the anti-malarial effect of TCM (artemisinin) which has also played an important role in effectively controlling COVID-19 in China. Simotang oral liquid (SMT) is a TCM prescription composed of four natural plants: Aucklandiae Radix, *Aurantii Fructus*, Arecae Semen, and Linderae Radix. The prescription of SMT was first proposed in 1519 and it was approved as an oral liquid drug by a manufacturer in 1994. It is widely used in China to treat gastrointestinal diseases such as abdominal distension, abdominal pain, diarrhea and constipation [7]. Though SMT has been used in curing gastrointestinal diseases in clinics for hundreds of years, there is still a lack of scientific research investigating the function of SMT. Chen *et al*. performed clinical research involving 90 patients to study the effect of SMT, and discovered that both oral administration of SMT and external use of glycerine enema were found beneficial to benign tumor patients for curing postoperative anal exhaust and anal spasm [8]. A meta-analysis of 2,713 patients suffering with functional dyspepsia (FD) also showed that SMT might be an effective and safe drug in the treatment of FD [9]. Cai *et al*. confirmed that SMT could promote gastrointestinal motility and treat FD by altering the level of serum motilin and the expression of cholecystokinin in mice [10]. Clinical studies were also performed to investigate the gastrointestinal protective effect of SMT on cancer patients who were suffering from side effects of radio- or chemotherapy. By treating 23 gastric cancer patients suffering chemotherapy after surgery with SMT, Wang *et al*. discovered that SMT could remarkably alleviate nausea, anorexia, vomiting, abdominal distension and constipation induced by chemotherapy [11]. In cervical cancer patients suffering side effects of neoadjuvant chemotherapy and concomitant IMRT radio-chemotherapy, SMT also exhibited reduction in nausea, vomiting, abdominal distension, and constipation, and relieved gastrointestinal radiation injury [12]. Another clinical study of cervical cancer patients also showed that patients treated with radiotherapy combined with endostar and paclitaxel might suffer nausea, vomiting, abdominal distension, constipation and radiation injury in gastrointestinal tract, and SMT could effectively protect the gastrointestinal function of these patients [13]. Although clinical trials have shown that SMT can be used to alleviate gastrointestinal side effects caused by chemotherapy, its mechanism is still unclear.

Studies have confirmed that oral TCM can directly or indirectly interact with gut microbiota and thereby affect the curative effect of TCM. On one hand, TCM might directly contract with gut microbiota and lead to promotion, inhibition, or elimination of microbiota [14]. On the other hand, TCM might change the gastrointestinal pH and gastrointestinal transit time to indirectly affect the composition of microbiota [14]. In addition, TCM could also adjust the immune system which might secrete enzyme and peptide that could regulate gut microbiota [14]. Therefore, altering gut microbiota is an important part of effective TCM treatment of diseases.

Moreover, the gut microbiota has been found to be involved in the pathogenesis of chemotherapy-induced gastrointestinal mucositis [15]. Sonis *et al*. classified the pathological process of the occurrence and development of chemotherapy-induced gastrointestinal mucositis into five stages: (1) initial stage, (2) signal upregulation and production stage, (3) signal amplification stage, (4) ulcer and (5) healing stage [16]. The gut microbiota plays a bidirectional regulatory function in the development of gastrointestinal mucositis. On the one hand, part of the gut microbiota binds to the Toll-like receptor family (TLRs), activating nuclear factor kappa B (NF-κB), which then promotes the development of inflammation. On the other hand, many commensal bacteria and their products can inhibit inflammation. For example, *Bifidobacterium* can increase the expression of tight junction proteins between intestinal epithelial cells, leading to a closer connection between mucosal epithelial cells, and protecting the continuity and integrity of the epithelial barrier [17]. Such findings indicate that gut microbiota could play important roles in regulating gastrointestinal side effects after chemotherapy treatment. Clinical trials have discovered that regulation of the gut microbiota could lead to a reduction in the intestinal toxicity caused by chemotherapy [18, 19]. Perales-Puchalt *et al*. discovered that the reconstitution of the microbiota can accelerate the healing of the intestinal epithelium and improve the systemic inflammatory response induced by DDP [20]. Therefore, it is necessary to study the mechanism by which SMT alleviates gastrointestinal side effects of chemotherapy by altering gut microbiota.

In our study, we focused on the protective effect of SMT against DDP-induced gastrointestinal mucositis and the underlying microbe-related mechanisms.

## Materials and Methods

### Reagents

Cisplatin (DDP, a commonly used drug in combination chemotherapy) was purchased from Dalian Meilun Biotechnology Co., Ltd. (China) and Simotang Oral Liquid (SMT) was purchased from Hunan Hansen Pharmaceutical Co., Ltd. (China). Antibiotics were purchased from Sigma-Aldrich (China) Trading Co., Ltd. Other chemicals and reagents used in this study were of analytical grade.

### Animals

Forty male BALB/c mice (5 weeks old), weighing 16 ± 2 g, were purchased from Beijing Weitong Lihua Co., Ltd. Animals were housed in cages of 5 mice per cage on a 12-h light/dark cycle with free access to rodent chow and sterile water. This experimental protocol was approved and implemented by the Experimental Animal Management Center of Jinan University (Animal Approval No. IACUC-20200927-01 and 20220114-16).

### Animal Experimental Protocol

Chemotherapy-induced gastrointestinal mucositis model: After one week of acclimatization, 20 male BALB/c mice were randomly divided into 4 groups: CTRL group, DDP group, DDP-SMT-LOW group, and DDP-SMT-HIGH group. CTRL group: intraperitoneal injection of 0.9% normal saline and sterile water by gavage daily. DDP group: intraperitoneal injection of DDP and sterile water by gavage daily. DDP-SMT-LOW group: intraperitoneal injection of DDP and low-dose SMT (5 ml/kg) by gavage daily. DDP-SMT-HIGH group: intraperitoneal injection of DDP and high-dose SMT by gavage (10 ml/kg) daily. Mice were intraperitoneally injected with DDP (2 mg/kg/day for 5 days) to establish a chemotherapy-induced gastrointestinal mucositis model [19].

Gut microbiota deletion model: After one week of acclimatization, 20 male BALB/c mice were randomly divided into 4 groups: CTRL group, ABX group, ABX+DDP group, and ABX+DDP+SMT group. From the first day until the end of the experiment, the ABX was added to the drinking water of the above three groups. The antibiotic-containing water was changed every 3 days and consisted of 4 antibiotics: ampicillin (1 g/l), neomycin (1 g/l), metronidazole (1 g/l) and vancomycin (0.5 g/l). Then on the fourth day, mice of ABX group, ABX+DDP group and ABX+DDP+SMT group were gavaged with an antibiotic cocktail (ABX) every day for 5 days to further deplete the intestinal flora. The ABX consists of 4 antibiotics: ampicillin (50 mg/ml), neomycin (50 mg/ml), metronidazole (50 mg/ml), and vancomycin (50 mg/ml). From the ninth day, mice of CTRL group and ABX group were intraperitoneally injected with 0.9% normal saline and sterile water by gavage daily. Mice of ABX+DDP group were intraperitoneally injected with DDP and sterile water by gavage daily. Mice of ABX+DDP+SMT group were intraperitoneally injected with DDP and SMT (10 ml/kg) by gavage daily.

### Sample Collection and Treatment

Body weight, food intake and water intake were recorded daily. After the experiment, the mice were anesthetized with isoflurane. Blood was extracted from the eyeballs and mice were sacrificed by cervical dislocation. Colon contents were collected immediately and placed in a cryopreservation tube, which was then transferred to a liquid nitrogen tank. The small intestine of mice was removed by laparotomy, the length of the small intestine was measured and photographed, and the ileum was cut into 3 parts approximately 1.0 cm in length. Two pieces of ileal tissue were collected and stored at -80°C for biochemical analysis.

### ELISA Assay of Blood Chemistry

Mouse blood was collected on the 6th day after DDP treatment. Blood GLU was measured using a GLU detection kit (Lei Du Life Science Co., Ltd., China).

An enzyme-linked immunosorbent assay (ELISA) kit (Solarbio Technology Co., Ltd., China) was used to determine the protein levels of IL-1β and IL-6 in serum.

### Histological Analysis

The tissue of the third ileum segment was fixed in 4% paraformaldehyde for 24 h, embedded in paraffin and sectioned. Haematoxylin-eosin (H&E) staining was used to observe the morphological changes. To quantitatively assess the extent of intestinal tissue damage, we used an Olympus BX53 fluorescence microscope and a video camera (magnification 20 ×) to observe the staining of ileal tissue and to take micrographs.

### Detection of Inflammatory Factor and Adhesive Factor Expression in Intestinal Tissues

The jejunum tissue stored at -80°C was removed, and the Total RNA Extraction Kit (Solarbio Technology Co., Ltd., China) was used to extract total RNA from 20 samples. Then, the Evo M-MLV RT Kit for qPCR (Hunan Accurate Biotechnology Co., Ltd., China) was used to synthesize cDNA. Finally, we used the SYBR Green Premix Pro Taq HS qPCR Kit II (Hunan Accurate Biotechnology Co., Ltd.) and qPCR technology to accurately quantify and detect the target genes of IL-1β, IL-6, claudin-2, occludin, and ZO-1 in small intestinal tissue.

### Primers

IL-1β-Forward: TGAGGACATGAGCACCTTC
IL-1β-Reverse: GGGAACGTCACACACCA
IL-6-Forward: ATGAGACTGGGGATGTCTGT
IL-6-Reverse: AAGGCAACTGGATGGAAGT
Occludin-Forward: CTGGATCTATGTACGGCTCACA
Occludin-Reverse: TCCACGTAGAGACCAGTACCT
ZO-1-Forward: GAGCGGGCTACCTTACTGAAC
ZO-1-Reverse: GTCATCTCTTTCCGAGGCATTAG
GAPDH-Forward: AAGAAGGTGGTGAAGCAGG
GAPDH-Reverse: GAAGGTGGAAGAGTGGGAGT

### 16S rRNA Amplicon Sequencing-Based Microbiota Community Analysis of Faecal Samples

Mouse feces samples were collected, intestinal flora RNA was extracted and isolated, and universal primers were used for PCR amplification. The MiSeq/HiSeq platform was used to sequence 16S rRNA. Alpha-diversity analysis was used to analyze the community diversity within each sample (within-community), and beta-diversity analysis was used for the comparative analysis of microbial community structure between different samples or groups (between-community).

### Statistical Analysis

SPSS 26.0 was used to carry out statistical analysis of the experimental data. The statistical methods utilized were univariate analysis of variance and the *t*-test. When *p* < 0.05, the experimental results were statistically significant (* for *p* < 0.05, * * for *p* < 0.01, * * * for *p* < 0.001); when p > 0.05, the experimental results were not statistically significant.

## Results

### SMT Effectively Alleviated the Gastrointestinal Side Effects Caused by DDP

**Fig. 1:**
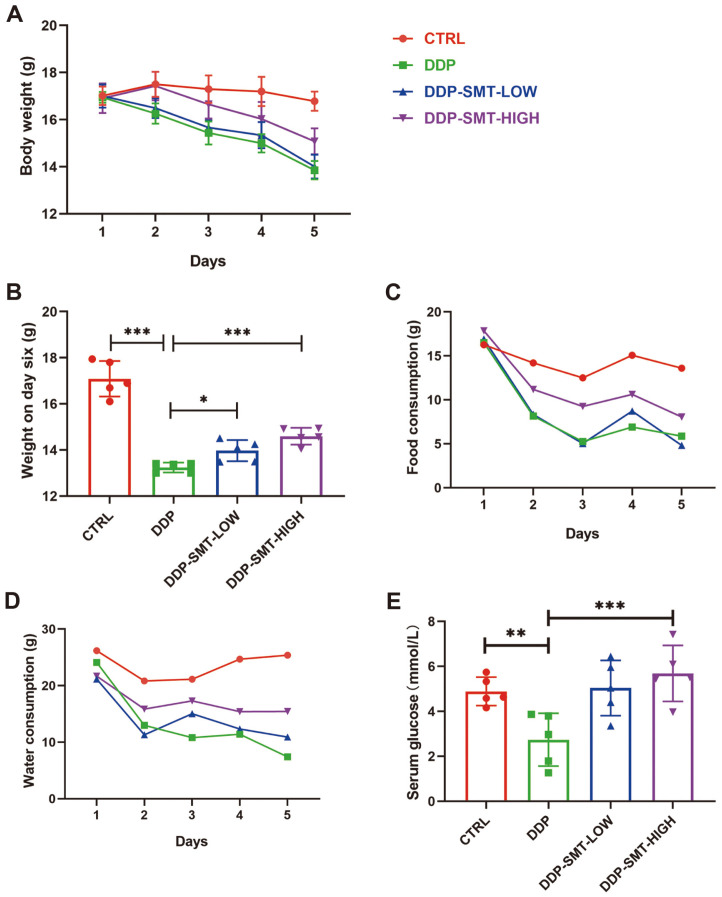
SMT alleviated the DDP-induced changes in body weight, food intake, water intake, and the decreased blood glucose in mice. **A**. Body weight of mice to evaluate the effect of DDP and SMT. Body weight was recorded prior to DDP injection daily. **B**. Body weight of mice on day six. **C** and **D**. Mouse food intake (**C**) and water intake (**D**) were recorded prior to DDP injection daily. **E**. Blood glucose levels of mice. Blood serum was collected and blood glucose was detected as described in the Materials and Methods.

Clinical research has shown that SMT could effectively alleviate gastrointestinal side effects induced by chemotherapy [11-13]. To demonstrate whether SMT could alleviate gastrointestinal side effects induced by chemotherapeutic drugs in animal models, we treated mice with cisplatin (DDP) and low/high doses of SMT. We found that DDP caused weight loss in mice during DDP treatment, while SMT effectively restored the DDP-induced loss of body weight (Figs. 1A and 1B). Food intake and water intake were also recorded. DDP was found to decrease food intake and water intake in mice, while SMT could restore the decrease in food intake and water intake caused by DDP, indicating that SMT could recover the appetite of DDP-treated mice (Figs. 1C and 1D). Mice serum was also collected at the end of the experiment, and we found that serum glucose levels were low in DDP-treated mice, which can damage the health of mice (Fig. 1E). SMT increased serum glucose levels in mice, indicating that the SMT-treated mice had healthier serum glucose levels (Fig. 1E).

**Fig. 2:**
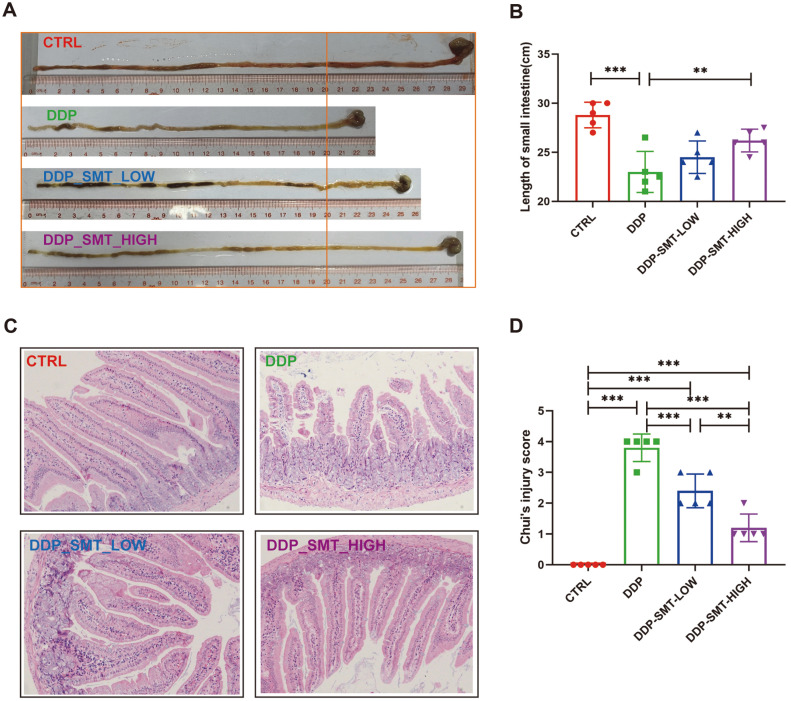
SMT relieves the damage induced by DDP to the small intestine. **A** and **B**. The length of the small intestine of the mice was photographed for comparison (**A**) and measured (**B**). **C**. H&E staining of the small intestine of mice was performed, and small intestinal mucosal epithelial cells, villi, apical mucosal ulcers, goblet cells, etc. were observed (Magnification 20×). **D**. Pathological score of small intestine tissue sections (small intestine Chui’s score).

The direct cytotoxic effect of chemotherapy on the gastrointestinal basal epithelial cells is the main mechanism leading to gastrointestinal mucositis. Cytotoxicity can cause damage to the structure and function of the gastrointestinal tract, such as the shortening of intestinal villi, ablation of intestinal crypts, accumulation of local inflammatory cells, impaired intestinal barrier function, and reduced intestinal digestive enzyme activity [16]. Mouse intestines were also collected and analyzed. The length of the small intestine of mice was shortened in the DDP group, while SMT was beneficial to the recovery of the length of the small intestine (Figs. 2A and 2B). H&E staining was performed on the small intestine tissue (jejunum), and we found that the small intestinal mucosa of the mice in the CTRL group had slender, high-tipped, intact, smooth, and neatly arranged villi, with evenly distributed goblet cells on the surface (Fig. 2C). The small intestine of the DDP-treated group showed an obvious inflammatory reaction: the small intestinal villi were shortened with oedema present, the top of the villi was ruptured, the intestinal crypts were ablated, the goblet cells were ruptured, and the number of goblet cells was also decreased (Fig. 2C). SMT effectively alleviated the damage caused by DDP to the small intestine (Fig. 2C). The Chui’s score was used to evaluate the degree of pathological damage to the small intestinal mucosa [21]. The Chui’s score in the DDP group was as high as 3.8, while the Chui's score in the SMT groups was significantly reduced by 34 and 68% (Fig. 2D).

**Fig. 3:**
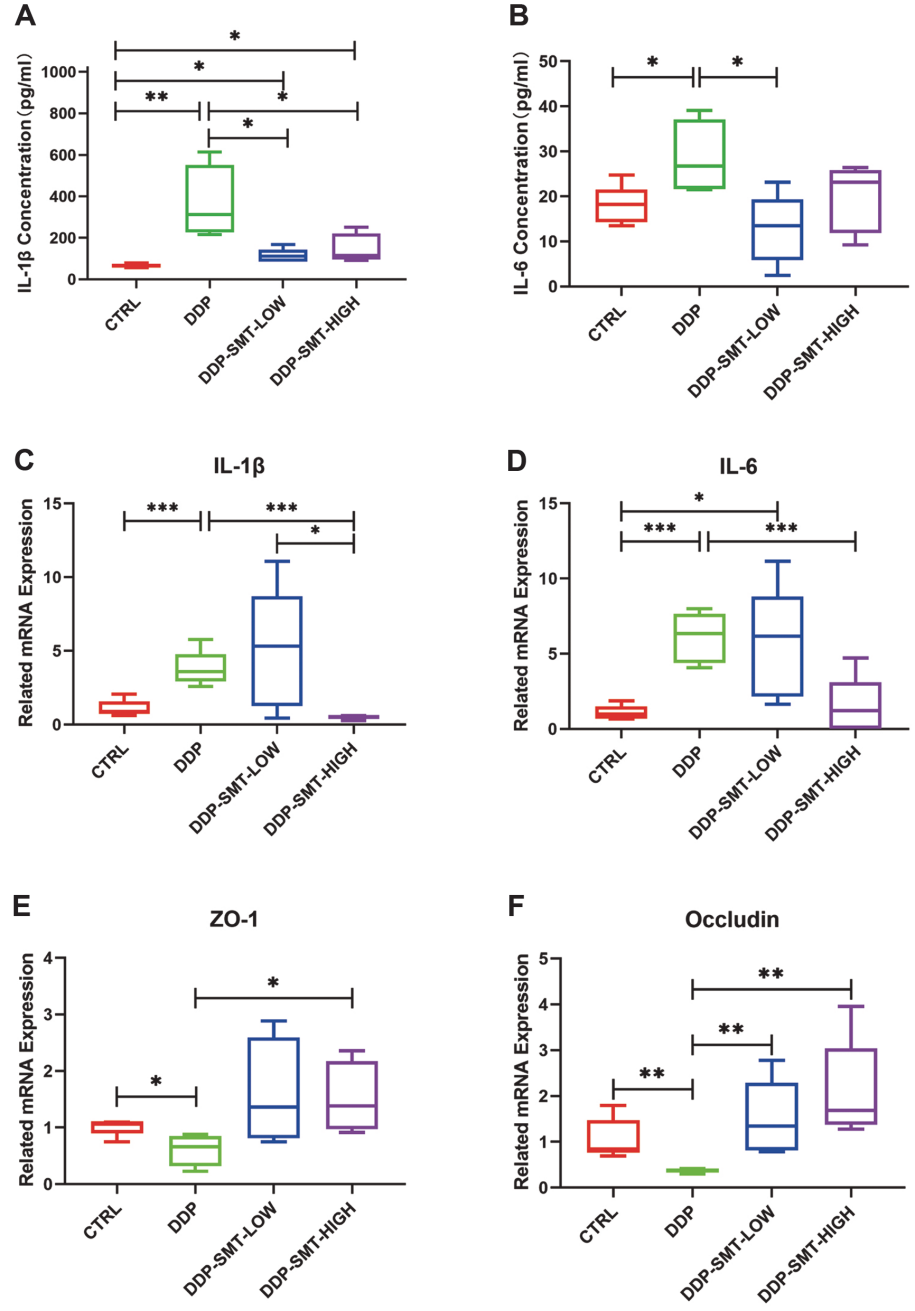
Effect of DDP and SMT on the expression of inflammatory factors and adhesive factors in mice. **A** and **B**. ELISA analysis of the levels of inflammatory factors IL-1β (**A**) and IL-6 (**B**) in mouse blood serum. **C** and **D**. Detection of the mRNA expression levels of the inflammatory factors IL-1β (**C**) and IL-6 (**D**) in mouse intestines. **E** and **F**. Detection of adhesive factor occludin (**E**) and ZO-1 (**F**) mRNA expression levels in mouse intestines.

Analysis of mouse intestines indicated that mice might suffer gastrointestinal mucositis after DDP treatment, and SMT could relieve the related inflammation. To determine changes in the inflammatory response in mice, concentration of inflammatory factor was also tested in vivo. By utilizing ELISA assay, the concentrations of IL-1β and IL-6 were found to increase in DDP-treated mouse serum compared to CTRL mouse serum (Figs. 3A and 3B). In addition, the concentrations of these two factors could be inhibited when treated with SMT, indicating remittance of inflammation in mice (Figs. 3A and 3B). The results of the ELISA assay show that both low and high doses of SMT could significantly reduce the expression of IL-1β. Low-dose SMT could significantly reduce IL-6, but the effect of high-dose SMT in altering IL-6 was not as good as low-dose SMT while the effect of SMT in altering IL-6 was not completely dependent of the dose of SMT. RNA was also extracted from mouse small intestine tissue (jejunum) to detect the expression of inflammation-related factors in the intestine. qPCR experiment results showed that the mRNA expression of inflammatory factors IL-1β and IL-6 was significantly increased in DDP-treated mice and that high-dose SMT can significantly reduce the expression of IL-1β and IL-6, but there was no statistical difference between the low-dose SMT group and the model group. (Figs. 3C and 3D). Interestingly, the concentration of IL-1β and IL-6 was different in mouse serum and small intestine tissue. This indicated that SMT might act first on the serum, then on the small intestine, and high-dose SMT might show better protective effects in reducing intestinal inflammation. The results above provide evidence that SMT could relieve gastrointestinal side effects caused by DDP in vivo.

Moreover, gastrointestinal mucositis induced by chemotherapy might also destroy the tight junction of intestinal epithelial cells [22], so the mRNA levels of the tight junction protein were also analyzed. Zonula occludens-1 (ZO-1) is an important component of tight junctions, and its downregulation or decreased activity affects the formation of tight junctions between cells [23]. Downregulation of occludin has also been found to cause damage to intestinal mucosal barrier function and to increase intestinal mucosal permeability [23]. The mRNA expression levels of ZO-1 and occludin were also detected. We also found that DDP could inhibited expression of ZO-1 and occludin, while low and high doses of SMT could effectively increase their mRNA expression (Figs. 3E and 3F). This finding indicated that DDP leads to the downregulation of these adhesive factors, which prevents the intestinal mucosa from exerting its important defense barrier function and increases the risk of harmful bacteria and toxins penetrating the intestine into the bloodstream and causing intestinal infections.

### DDP and SMT Could Change the Structure of the Gut Microbiota in Mice

**Fig. 4:**
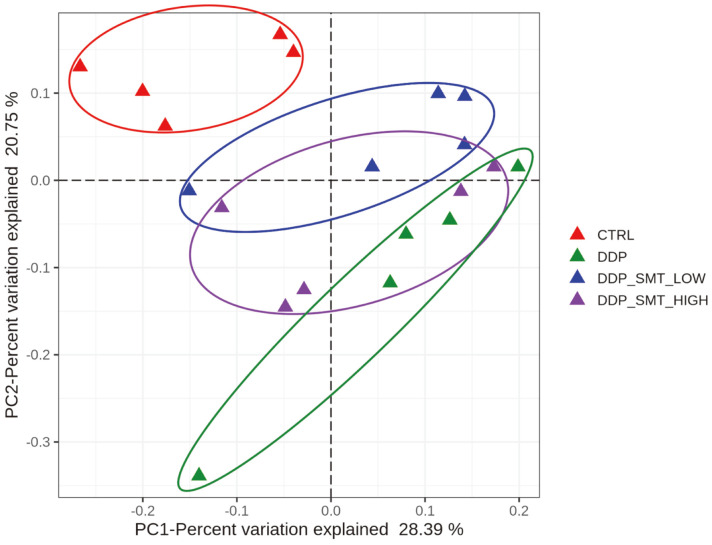
PCoA weighted UniFrac analysis of mouse gut microbiota. Principal co-ordinates analysis (PCoA) can be used to characterize the evolutionary similarities and differences in the composition of microbial communities. It showed that the microbiota of the DDP groups was significantly separated from the normal group and the SMT group.

Gut microbiota has been found to play important roles in gastrointestinal mucositis caused by chemotherapy [17]. Therefore, we wondered whether SMT could regulate gut microbiota homeostasis, thereby alleviating the gastrointestinal side effects caused by DDP. To find out, we performed 16S rDNA amplicon sequencing and the alpha-diversity analysis results showed that both the DDP and SMT groups did not have significant changes compared to the CTRL group, showing that there was little difference in the alpha-diversity of the flora in each sample (data not shown). Although the alpha-diversity analysis showed little difference, the beta-diversity analysis revealed some significant changes in different groups. The PCoA weighted UNIFRAC analysis showed that the distribution of intestinal microflora in the DDP group was significantly changed, while the low- and high-dose SMT could reverse the change in intestinal microflora caused by DDP (Fig. 4). This finding indicated that DDP might change the abundance of different gut microbiota species compared to the CTRL group, and SMT might reverse the changes induced by DDP.

### SMT Reversed the DDP-Induced Changes at the ‘Phylum’ Level of Gut Microbiota

**Fig. 5:**
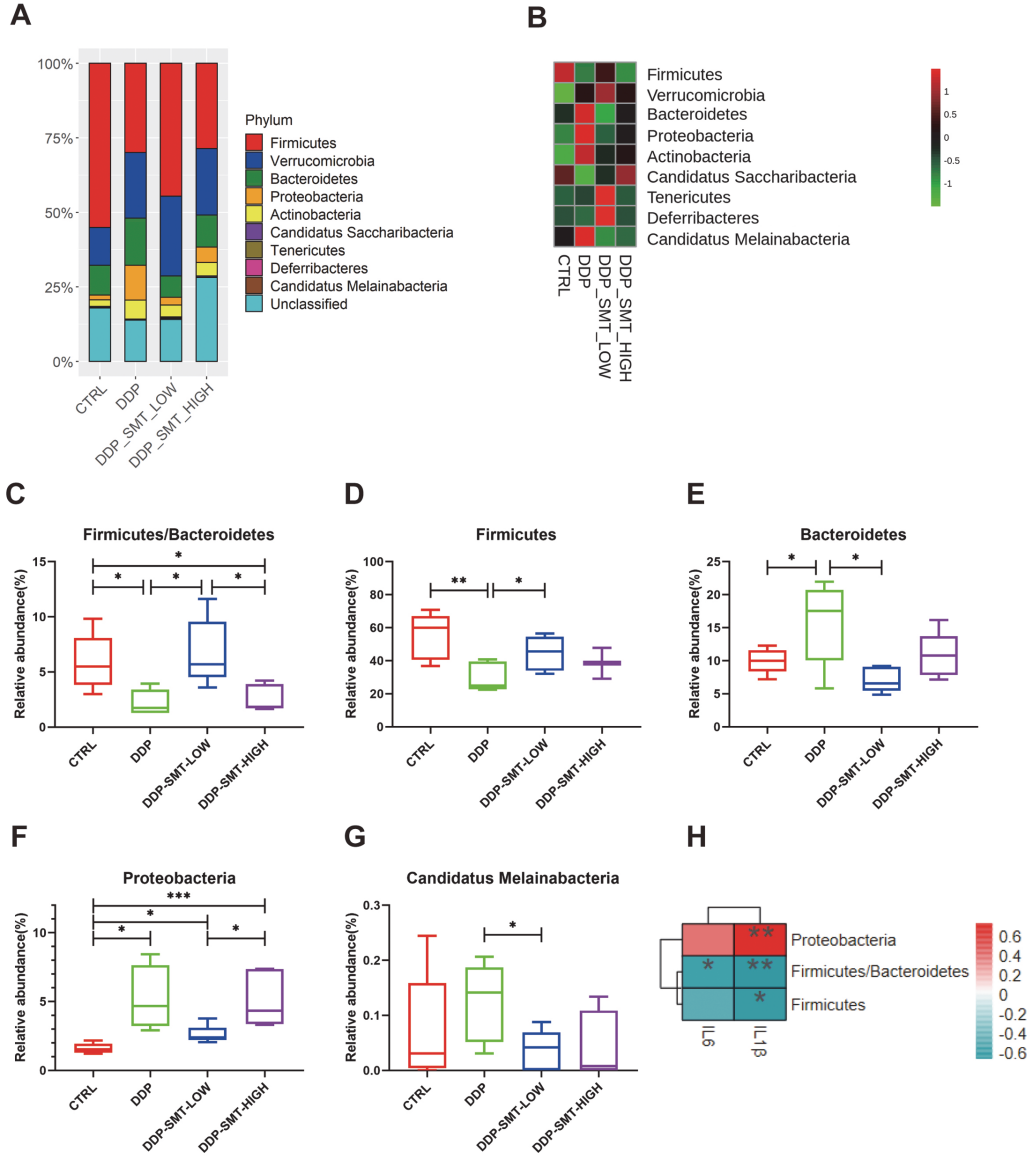
SMT reversed the ‘phylum’ level changes in gut microbiota caused by DDP. **A**. Stackbar histogram of the distribution of bacterial phyla in the gut microbiota of mice (‘phylum’ level). **B**. Heatmap of the phylum abundance of mouse gut microbiota. **C-G**. Analysis diagram of the abundance of each group of mouse gut microbiota. Among them, **C** is the ratio of Firmicutes/Bacteroidetes, **D** is the abundance of Firmicutes, **E** is the abundance of Bacteroidetes, **F** is the abundance of Proteobacteria, and **G** is the abundance of *Candidatus Melainabacteria*. **H**. Heatmap of correlations between phylum-level gut microbiota and serum inflammatory factors. Negative correlations are shown in blue, and positive correlations are shown in red. The darker the color, the higher the correlation.

Since the beta-diversity analysis showed changes between the DDP- and SMT-treated mice, we then analyzed the abundance of specific flora at the ‘phylum’ classification level of gut microbiota to identify important phyla of microbiota regulated by DDP and SMT. We found that the gut microbiota of mice contained many different phyla, among which Firmicute, Verrucomicrobia, Bacteroidetes, Proteobacteria and Actinobacteria had the highest abundance (Figs. 5A and 5B).

It has been reported that the ratio of Firmicutes/Bacteroidetes is closely related to intestinal inflammatory disease and that patients with a low Firmicutes/Bacteroidetes ratio may be considered to be suffering from intestinal inflammatory disease [24]. Our study showed that DDP could significantly reduce the ratio of Firmicutes/Bacteroidetes in the intestines of mice, suggesting that DDP might induce intestinal inflammation in mice (Fig. 5C, Table S1). SMT restored the ratio of Firmicutes/Bacteroidetes in the intestines of mice to play a role in reducing gastrointestinal inflammation (Fig. 5C, Table S1).

Furthermore, Firmicutes have also been reported to be associated with inflammation, and their abundance can be reduced in mice with chronic autoimmune arthritis [25]. Our research discovered that DDP downregulated the abundance of Firmicutes in the intestines of mice, and SMT effectively increased the level of Firmicutes in mice (Fig. 5D, Table S1). Delday *et al*. discovered that the relative abundance of Bacteroides was reduced in inflammatory bowel diseases (such as Crohn's disease) [26]. Our study also found that the abundance of Bacteroidetes was elevated in the DDP-treated mice, and SMT reduced their relative abundance, thereby alleviating intestinal inflammation. (Fig. 5E, Table S1). Proteobacteria are also one of the flora that is closely related to intestinal inflammation [27]. Studies have found that the increased abundance of Proteobacteria was related with UC in mice [28]. We found that DDP significantly increased their abundance in the intestines of mice (Fig. 5F, Table S1). Although SMT cannot significantly reduce the abundance of Proteobacteria, it can reduce their expression to a certain extent (Fig. 5F, Table S1). Furthermore, although there is still a lack of research investigating the function of *Candidatus Melainabacteria*, our study found that their abundance in the small intestine of mice was also decreased in SMT-treated mice compared to DDP-treated mice (Fig. 5G, Table S1). The differences in the above bacterial phyla indicate that DDP can cause changes in the abundance of intestinal inflammation-related bacterial phyla, thereby causing intestinal inflammation. SMT can treat intestinal inflammation by altering the abundance of related bacterial phyla.

In addition, researchers also found that some of the bacteria that can produce butyric acid are concentrated in Firmicutes [29]. Butyrate, a product of the intestinal flora, has been found to improve the therapeutic effect of tumor drugs [30]. Our results show that DDP can reduce the abundance of Firmicutes while SMT can increase their level (Fig. 5D, Table S 1). The results show that SMT can also increase the abundance of the butyric acid-producing Firmicutes bacteria, thereby improving the therapeutic effect of tumor drugs.

The correlation between gut microbiota at the phylum level and inflammatory factors (IL-1β, IL-6) in serum was also analyzed (Fig. 5H). The results showed that concentration of IL-1β was negatively correlated with abundance of Firmicutes, the ratio of Firmicutes/Bacteroidetes, and positively correlated with abundance of Proteobacteria. Concentration of IL-6 was also negatively correlated with the ratio of Firmicutes/Bacteroidetes. These findings indicated that these three bacterial phyla have significant correlations with the secretion of inflammatory factors (IL-1β, IL-6) and the secretion of IL-1β seems to be more dependent on the regulation of gut microbiota compared to IL-6.

### SMT Could Reduce the Side Effects of DDP and Enhance the Curative Effect by Adjusting the Abundance of Probiotics and Anticancer Bacteria at the ‘Genus’ Level

**Fig. 6:**
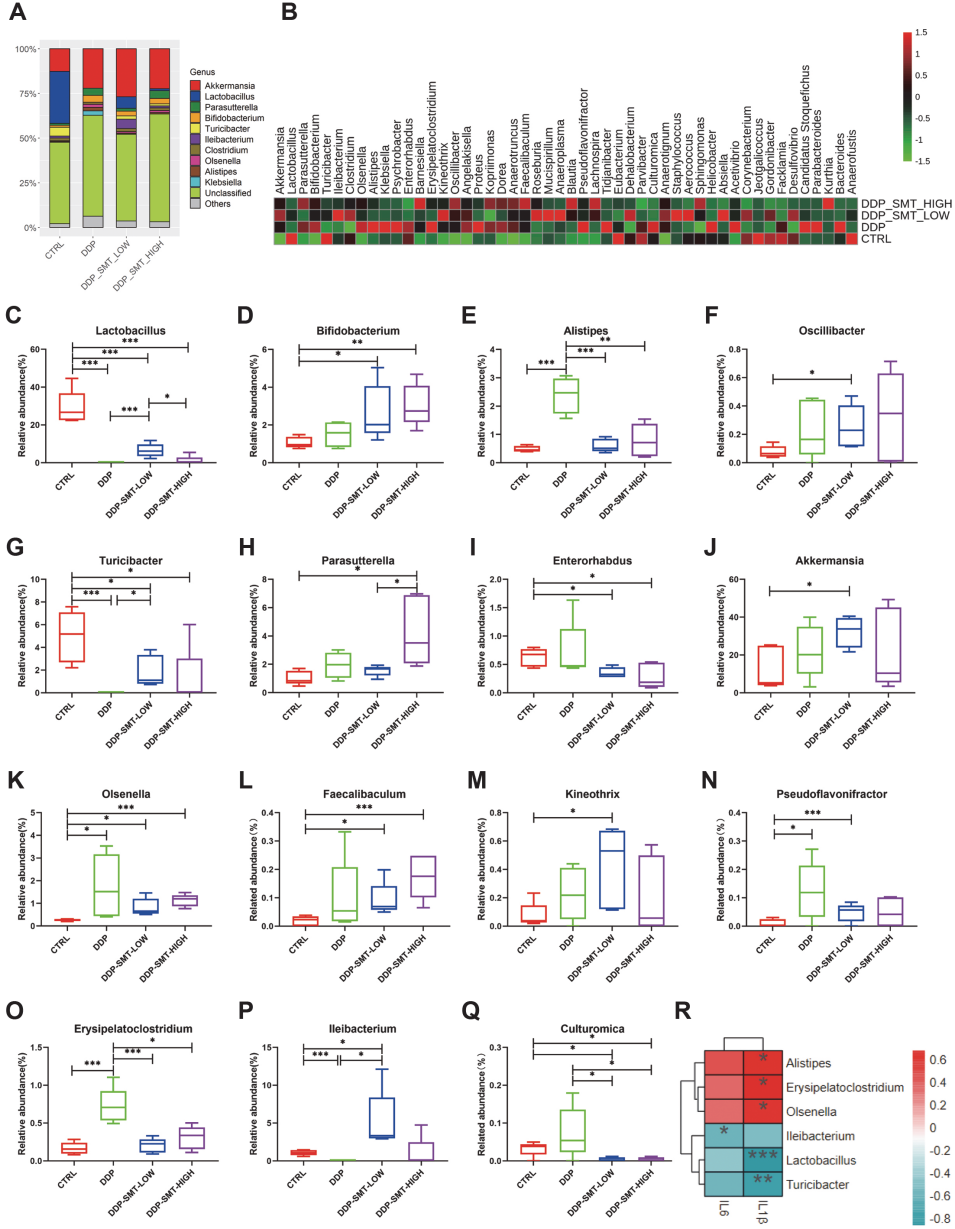
SMT reduced the side effects of DDP and enhanced the efficacy of DDP by altering the gut microbiota at the ‘genus’ level. **A**. Stackbar histogram of the distribution of bacteria in the gut microbiota of mice (‘genus’ level). **B**. Heatmap of the genus abundance of bacteria in the gut microbiota of mice. **C-Q**. Analysis diagram of the abundance of each group of mouse gut microbiota. **C** is the *Lactobacillus* abundance, **D** is the *Bifidobacterium* abundance, **E** is the *Alipites* abundance, **F** is the *Oscillibacter* abundance, **G** is the Akkermansia abundance, **H** is the *Olsenella*, **I** is the *Faecalibaculum* abundance, **J** is the *Kineothrix* abundance, **K** is the *Turicibacter* abundance, **L** is the *Parasutterella* abundance, **M** is the *Erysipelatoclostridium* abundance, **N** is the *Ileibacterium* abundance, **O** is the *Pseudoflavonifractor* abundance, **P** is the *Culturomica* abundance, and **Q** the is *Enterorhabdus* abundance. R. A heatmap of correlations between genus-level gut microbiota and serum inflammatory factors. Negative correlations are shown in blue, and positive correlations are shown in red. The darker the color, the higher the correlation.

Apart from the ‘phylum’ classification level of gut microbiota, the ‘genus’ level of microbiota was also analyzed. We discovered that both DDP and SMT caused significant changes in the abundance of different genera of mouse intestinal flora (Figs. 6A and 6B). *Lactobacillus* and *Bifidobacterium* are two common probiotics and studies have shown that they both have anti-inflammatory effects [31-33]. Our research found that DDP can significantly reduce the abundance of *Lactobacillus*, while SMT can restore the level of *Lactobacillus* (Fig. 6C, Table S2). Moreover, although DDP did not have a significant effect on the abundance of *Bifidobacterium*, SMT also increased the abundance of *Bifidobacterium* (Fig. 6D, Table S2). This indicates that SMT could effectively increase the abundance of probiotics in the body, thereby curing intestinal inflammation. *Alistipes* has been proven to be a pathogen of colorectal cancer and is closely related to intestinal inflammation [34]. We found that DDP significantly increased the abundance of *Alistipes*, while SMT effectively reduced its abundance (Fig. 6E, Table S2). *Oscillibacter*, another intestinal bacterial genus with anti-inflammatory effects [35], was also found to be highly expressed in SMT-treated mice (Fig. 6F, Table S2). *Turicibacter*, a flora that was reduced in inflammatory bowel disease, oral mucosal ulcers and other inflammations [36, 37], was found to be suppressed by DDP, while SMT restored its abundance to a certain extent (Fig. 6G, Table S2). *Parasutterella* participated in cholesterol metabolism and could cause elevated levels of hypoxanthine which plays a beneficial role in protecting the homeostasis of the intestinal mucosa [38]. In mice treated with high doses of SMT, the abundance of *Parasutterella* was also increased (Fig. 6H, Table S2). Zhao *et al*. discoverer that the abundance of *Enterorhabdus* decreased with the alleviation of the inflammatory response induced by a high-fat and high-fructose diet [39]. Our results also showed that SMT could reduce the abundance of *Enterorhabdus* and may help relieve intestinal inflammation (Fig. 6I, Table S2). These results indicate that SMT can reduce the gastrointestinal side effects of chemotherapy drugs by inhibiting inflammation-related bacteria and increasing the abundance of anti-inflammatory bacteria.

Not only was the abundance of inflammation-related bacteria changed by DDP and SMT, the abundance of bacteria that exhibited anticancer ability could also be regulated. *Akkermansia* is a famous anticancer bacterium that has been found to improve the therapeutic effect of tumor drugs in a variety of tumors [40, 41]. Our results indicated that SMT could increase the abundance of *Akkermansia*, thereby enhancing the anticancer efficacy of DDP (Fig. 6J, Table S2). *Olsenella* and *Faecalibaculum* have also been reported to be two anticancer bacteria species that can improve tumor treatment effects [42, 43]. Our research showed that DDP could increase the abundance of *Olsenella*, while SMT can effectively increase the abundance of both *Olsenella* and *Faecalibaculum* (Figs. 6K and 6L, Table S2). *Kineothrix*, a genus of butyric acid-producing bacteria [44], was also overexpressed in SMT-treated mice (Fig. 6M, Table S2). *Pseudoflavonifractor*, another probiotic that can produce butyric acid and regulate immune function [45], was found to increase by both DDP and SMT (Fig. 6N, Table S2). The above results indicated that SMT could increase the abundance of anticancer bacteria and butyric acid-producing bacteria to increase the therapeutic effect of chemotherapy drugs.

In addition to inflammation-related and antitumor-related flora, we also found that DDP and SMT can regulate other flora. For example, DDP was found to increase the abundance of harmful bacterium *Erysipelatoclostridium* [46], while SMT inhibited its level, indicating that SMT can reduce the abundance of harmful bacteria to reduce the toxicity of DDP (Fig. 6O, Table S2). Moreover, *Ileibacterium*, a flora that was positively correlated with atherosclerosis [47], were also found to be reduced by DDP, while SMT restored its expression (Fig. 6P, Table S2). There are few reports focusing on the function of *Culturomica*. However, we found that *Culturomica* and *Alistipes* belong to the same phylum, Bacteroides, and the trend in the murine gastrointestinal mucositis model is the same (increased abundance might promote ileitis) [48], and SMT could effectively reduce the abundance of *Culturomica* (Fig. 6Q, Table S2).

The correlation heatmap was used to visualize the correlation between gut microbiota at the genus level and inflammatory factors (IL-1β, IL-6) in serum (Fig. 6R). Whether at the genus or phylum level, the concentration of IL-1β and IL-6 showed a similar trend in association with gut microbiota. We found that the concentration of IL-1β was positively correlated with abundance of *Alistipes*, *Erysipelatoclostridium* and *Olsenella*, and negatively correlated with abundance of *Lactobacillus* and *Turicibacter*. Concentration of IL-6 was also negatively correlated with abundance of *Ileibacterium*.

### LEfSe Analysis of Gut Microbiota Sequencing

**Fig. 7:**
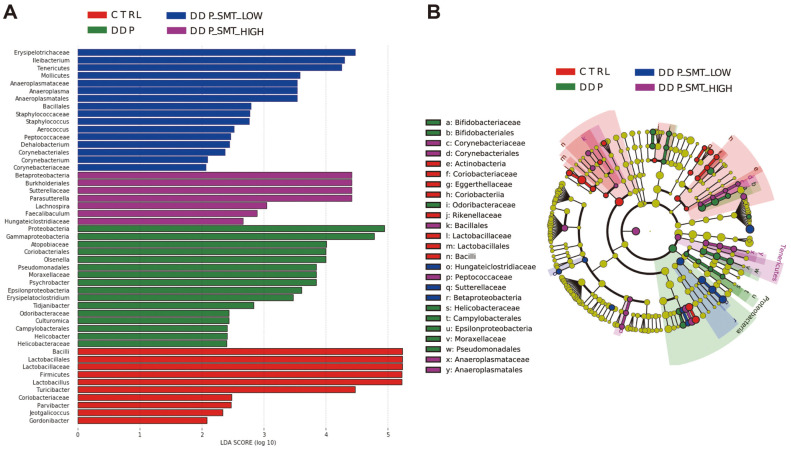
LEfSe analysis of gut microbiota sequencing. **A**. LDA value distribution histogram. The ordinate is the name of the bacterial species with a significant difference in abundance, and the abscissa is its corresponding LDA value. The higher the LDA value, the more obvious the difference is between it and other groups. **B**. Species evolutionary branch diagram. Different nodes represent different bacterial species, the size of the node represents the abundance of bacterial species, and the circle from inside to outside represents kingdom to genus (kingdom-phylum-class-order-family-genus) classification of bacterial species. Yellow represents the bacteria with no difference, red represents the bacteria with the highest expression abundance and significant difference in the CTRL group, green represents the bacteria with the highest expression abundance and significant difference in the DDP group, blue represents the bacterial species with the highest expression abundance and significant difference in the DDP-SMT-LOW group, and purple represents the bacterial species with the highest expression abundance and significant difference in the DDP-SMT-HIGH group.

Moreover, LEfSe analysis was used to compare gut microbiota with significant differences in abundance between the four groups and their evolution process (Fig. 7). Among them, Firmicutes (phylum), Bacilli (class), Lactobacillales (order), *Lactobacillaceae* (family), *Lactobacillus* (genus), and *Turicibacter* (genus) in the CTRL group had the highest abundance and significant differences (Fig. 7). In the DDP group, Proteobacteria (phylum), Gammaproteobacteria (class), Coriobacteriales (order), *Atopobiaceae* (family), and *Olsenella* (genus) had the highest abundance and significant differences (Fig. 7). *Erysipelotrichaceae* (family), *Ileibacterium* (genus), Tenericutes (phylum), Mollicutes (class), *Anaeroplasma*tales (class), *Anaeroplasmataceae* (family), and *Anaeroplasma* (genus) in the DDP-SMT-LOW group had the highest abundance and significant differences (Fig. 7). Betaproteobacteria (class), Burkholderiales (order), *Sutterellaceae* (family), and *Parasutterella* (genus) in the DDP-SMT-HIGH group had the highest abundance and significant differences (Fig. 7).

### Gut Microbiota Is Indispensable for SMT in Improving the Gastrointestinal Side Effects Caused by DDP

**Fig. 8:**
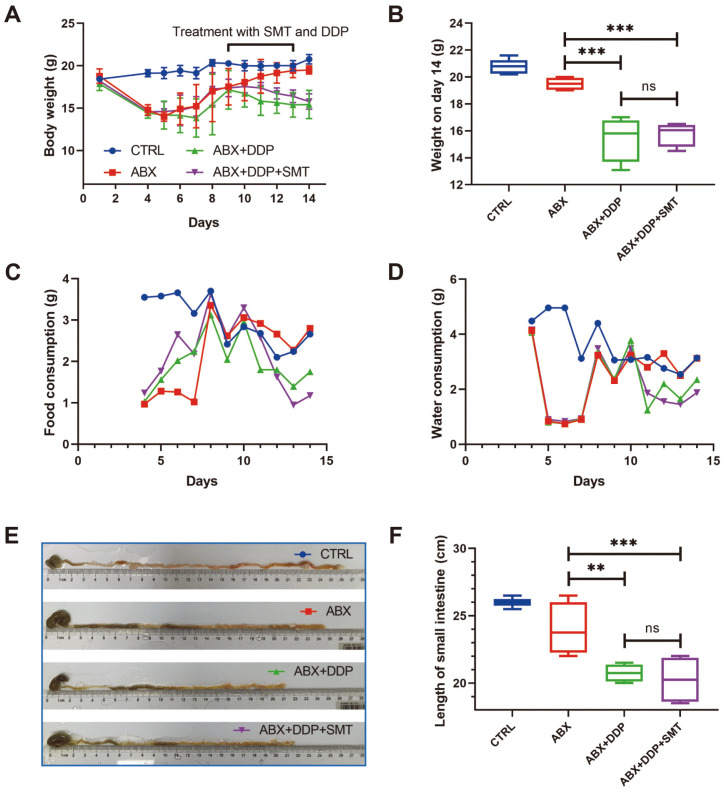
SMT was unable to cure DDP-induced gastrointestinal side effects in the ABX-treated mouse model. **A**. The body weight of mice reflects the effects of DDP and SMT. Body weights were recorded on the first day, and every day after two days. **B**. Body weight of mice on day 14. **C** and **D**. Mice food intake (**C**) and water intake (**D**) were recorded on the first day, and every day after two days. **E** and **F**. The length of the small intestine of the mice was photographed for comparison (**E**) and measured (**F**).

The results above indicated that gut microbiota might be important in the regulation of gastrointestinal side effects by SMT. To demonstrate the role of gut microbiota in SMT, we treated mice with a cocktail of antibiotics (ABX) to delete gut microbiota in mice. We discovered that the body weights of mice treated with ABX dropped sharply in the first three days compared to the Ctrl group of mice, indicating that mice were not adapted to antibiotics when they were first exposed to them (Fig. 8A). From day 4 to day 8, mice were gavaged with ABX but their body weight slowly recovered. And at the end of the experiment, the body weight of the ABX group recovered to normal compared to the CTRL group, demonstrating that the mice were adapting to the effects of the antibiotic (Fig. 8A). From day 9, mice of the ABX+DDP group and the ABX+DDP+SMT group were treated with DDP and DDP+SMT, and we found that mice of these two groups lost weight again (Fig. 8A). On day 14, the body weight of DDP-treated mice was found significantly lower than CTRL and ABX groups (Fig. 8B). In addition, there was no significant difference in body weight between the ABX+DDP group and the ABX+DDP+SMT group mice, indicating that in the ABX-treated mouse model, SMT could not alter the weight loss caused by DDP (Fig. 8B). Food and water intake of mice was also measured, revealing that in the ABX-treated mouse model, DDP reduced food and water intake in mice, while SMT could not restore DDP-induced reductions in food and water intake (Figs. 8C and 8D). Moreover, the small intestine of mice was also collected and measured. The length of the small intestine of mice in the ABX+DDP group and the ABX+DDP+SMT group was shortened, and there was no statistical difference between the two groups, showing that in the ABX-treated mouse model, SMT could not restore DDP-induced shortening of small intestine length (Figs. 8E and 8F). These results indicated that gut microbiota is indispensable for SMT treatment in alleviating gastrointestinal side effects.

## Discussion

The incidence of malignant tumors in human diseases is increasing, but side effects caused by chemotherapy drugs have received little attention. DDP is one of the most often used drugs in current chemotherapy, and we discovered that DDP could lead to intestinal flora imbalance, which then induces intestinal mucositis. Moreover, the mechanism by which SMT alleviates the gastrointestinal side effects caused by chemotherapy by regulating gut microbiota was explored. Our results showed that SMT could regulate the gut microbiota and maintain the normal structure and function of the intestinal mucosa, which led to the improvement of gastrointestinal side effects caused by DDP.

Most of the TCM was taken orally, and gut microbiota might be affected by the drugs and then further influenced the therapeutic effect of TCM. Existing research has confirmed that Chinese herbs (*Poria cocos* and its components) can regulate gut microbiota, thereby protecting against intestinal injury caused by DDP [19]. Our experiments showed that SMT also had a similar mechanism. The structure and distribution of the gut microbiota of mice in the DDP group were found to change significantly, and the low- or high-dose SMT group reversed these changes, which then alleviated the intestinal injury. We also discovered that SMT could recover the abundance of gut microbiota changes induced by DDP. By analyzing abundance of gut microbiota, SMT was found to reduce the abundance of inflammation-related bacteria and increase the abundance of anti-inflammatory bacteria. These results suggested that SMT could alleviate the intestinal inflammation caused by DDP by altering gut microbiota. Furthermore, anti-cancer bacteria were also analyzed. SMT was found to enhance the abundance of anticancer bacterium and butyric acid-producing bacteria. This finding indicated that SMT could increase the efficacy of chemotherapeutic drugs by altering gut microbiota.

To further validate the importance of gut microbiota in SMT improving DDP-induced gastrointestinal side effects, antibiotics were used to delete gut microbiota in mice. We discovered that SMT could not restore DDP-induced gastrointestinal side effects without gut microbiota. Once again the findings demonstrated that gut microbiota is an important mediator of SMT to alleviate the adverse changes caused by DDP.

Apart from changing of gut microbiota, gastrointestinal mucositis is another common phenotype induced by chemotherapy. We also observed that DDP could induce gastrointestinal mucositis in C57BL/6J mice. Expression of inflammatory factors was also tested and we found that while DDP enhanced the expression of the proinflammatory factors IL-1β and IL-6, SMT effectively reduced the DDP-induced mRNA expression of IL-1β and IL-6. Our results also showed that the levels of IL-1β and IL-6 were significantly negatively correlated with the Firmicutes/Bacteroidetes ratio. In addition, the concentration of IL-1β was also significantly negatively correlated with the abundance of *Firmicutes*, *Lactobacillus*, *Turicibacter*; and significantly positively correlated with the abundance of Proteobacteria, *Alistipes*, *Erysipelatoclostridium*, and *Olsenella*. Moreover, the concentration of IL-6 was negatively correlated with the abundance of *Ileibacterium*. Therefore, we speculate that SMT may improve the integrity and function of the intestinal barrier by altering gut microbiota, which thereby reduces the invasion of toxins and bacteria and reduces intestinal inflammation.

By analyzing the components in SMT and their relationship with gut microbiota and inflammation, we discovered some SMT key components that might be important in regulating gut microbiota. Studies have proven that five of the components in *Aurantii Fructus* (naringenin, hesperetin, hesperidin, neohesperidin, tangeretin) and a component in Arecae Semen (chlorogenic acid) have the ability to regulate gut microbiota [49-54]. Combined with our experimental data, we speculated that these six ingredients might be key components in SMT to relieve gastrointestinal side effects. For example, abundance of Bacteroidetes could be inhibited by naringenin, neohesperidin, tangeretin and chlorogenic acid, while neohesperidin, tangeretin and chlorogenic acid increased the Firmicutes abundance, resulting in the enhancement of Firmicutes/Bacteroidetes ratio and relief of inflammation. The abundance of two probiotics (*Lactobacillus* and *Bifidobacterium*) might be induced by all the six ingredients, and tangeretin was able to reduce the abundance of *Alistipes*. Besides, hesperidin has been found to induce the abundance of butyric acid-producing bacteria, indicating hesperidin might help improve the therapeutic effect of antitumor drugs by regulating gut microbiota. Apart from the six ingredients, there might be other effective SMT components to be further studied. Our results only revealed a small part of the mechanism how SMT improves gastrointestinal side effects induced by chemotherapy. There is still a need for further research to obtain a clearer understanding of this mechanism.

In conclusion, our study showed that SMT may reverse the imbalance of the gut microbiota and alleviate the gastrointestinal mucositis caused by DDP. Moreover, our results suggested that SMT can be used to treat intestinal injury caused by DDP chemotherapy. TCM provides a new drug choice with less toxicity for alleviating the side effects of chemotherapy.

## Supplemental Materials

Supplementary data for this paper are available on-line only at http://jmb.or.kr.
