# Supplementary material for: Simotang Alleviates the Gastrointestinal Side Effects of Chemotherapy by Altering Gut Microbiota
Source: J Microbiol Biotechnol. 2022 Feb 28;32(4):405–18. doi: 10.4014/jmb.2110.10018 (PMC9628794; doi:10.4014/jmb.2110.10018)
Supplement: Supplementary file 1 [file jmb-32-4-405-supple.pdf]

J. Microbiol. Biotechnol.
https://doi.org/10.4014/jmb.2110.10018

Supplemental Table 1. SMT reversed the DDP-induced changes at the 'phylum' level of gut microbiota

Name	DDP Group	SMT Groups	Effect
The Ratio of Firmicutes/Bacteroidetes	DOWN	UP	Patients with a low ratio of Firmicutes/Bacteroides may have intestinal inflammatory diseases.
Firmicutes	DOWN	UP	Low abundance of Firmicutes is related to inflammation.
Bacteroidetes	UP	DOWN	Abundance of Bacteroides is enhanced in ulcerative colitis.
Proteobacteria	UP	UP	Increased abundance of Proteobacteria is related with ulcerative colitis.
Candidatus Melainabacteria	UP	DOWN	SMT can reduce the abundance of Candidatus Melainabacteria.

Supplemental Table 2. SMT reversed the DDP-induced changes at the 'genus' level of gut microbiota

NAME	BELONGING (PHYLUM)	DDP GROUP	SMT GROUPS	EFFECT
Lactobacillus	Firmicutes	DOWN	UP	Lactobacillus (probiotic) has anti-inflammatory and anti-cancer effects on colorectal cancer associated with colitis.
Bifidobacterium	Actinobacteria	--	UP	Bifidobacteria (probiotic) can strengthen the intestinal mucus layer and relieve intestinal inflammation.
Alistipes	Bacteroides	UP	DOWN	Alistipes is one of pathogenic bacteria in colorectal cancer and promotes ileal inflammation.
Oscillibacter	Firmicutes	--	UP	Abundance of Oscillibacter is increased in ulcerative colitis.
Turicibacter	Firmicutes	DOWN	UP	Abundance of Turicibacter is significantly reduced in inflammation such as inflammatory bowel disease and oral mucosal ulcers.
Parasutterella	Proteobacteria	--	UP	Parasutterella causes increased levels of hypoxanthine (helps to protect the homeostasis of the intestinal mucosa) and has anti-infection effects.
Enterorhabdus	Actinobacteria	--	DOWN	The abundance of Enterorhabdus decreased with the alleviation of the inflammatory response induced by the high-fat and high-fructose diet.
Akkermansia	Verrucomicrobia	--	UP	Anti-cancer star bacteria, and its abundance is reduced in patients with

				cancer.
Olsenella	Actinobacteria	UP	UP	Olsenella can enhance the anti-tumor ability of T cells in a variety of tumor.
Faecalibaculum	Firmicutes	--	UP	Faecalibaculum exert anti-tumor effects by producing short-chain fatty acids.
Kineothrix	Firmicutes	--	UP	Kineothrix can produce butyric acid. Butyric acid enhances the immune barrier of the intestinal mucosa and has anti-inflammatory and anti-cancer effects.
Pseudoflavonifractor	Firmicutes	UP	UP	Pseudoflavonifractor is a probiotic that can produce butyric acid and regulate immune function.
Erysipelatoclostridium	Firmicutes	UP	DOWN	Erysipelatoclostridium is a conditional pathogen that can invasively infect patients with weakened immune function.
Ileibacterium	Firmicutes	DOWN	--	Ileibacterium is positively correlated with atherosclerosis.
Culturomica	Bacteroides	UP	DOWN	Culturomica belongs to the family Porphyromonadaceae (increased in arthritis and ileitis). Culturomica and Alitipes belong to the same phylum Bacteroides, and the trend is the same (increased abundance might promote ileitis).
